# Association between use of nutrition labels and healthy eating efforts before and after labeling policy changes in Mexico, 2019–2021

**DOI:** 10.1016/j.pmedr.2026.103624

**Published:** 2026-09-12

**Authors:** Cecilia I. Oviedo-Solís, Kathia L. Quevedo, Alejandra Jáuregui, Alejandra Contreras, Lana Vanderlee, Rachel E. Davis, James F. Thrasher, David Hammond

**Affiliations:** a Nutrition and Health Research Center, National Institute of Public Health. Av. Universidad 655, Col Santa María Ahuacatitlán, 62100, Cuernavaca. Mexico; b Leibniz Institute for Prevention Research and Epidemiology – BIPS. Achterstraße 30, 28359 Bremen, Germany; c School of Nutrition, Laval University. 2425 Rue de l'Agriculture, Québec, QC G1V 0A6, Canada; d Department of Health Promotion, Education and Behavior, Arnold School of Public Health, University of South Carolina, 915 Greene Street, Discovery I, Columbia, SC 29208, USA; e School of Public Health and Sciences, University of Waterloo, 200 University Ave West, Waterloo, ON N2L 3G1, Canada

**Keywords:** Nutrition labels, Warning labels, Guideline daily amounts, Nutrition fact tables, Mexico, Adults, Healthy eating efforts

## Abstract

**Objective:**

To examine associations between self-reported use of Nutrition Fact Tables (NFTs) and front-of-package nutrition labels (FoPNLs) with self-reported healthy eating efforts, and to assess whether these associations became stronger after Mexico replaced Guideline Daily Amounts (GDA) with Warning Labels (WL).

**Methods:**

Data come from the 2019 and 2021 Mexico administration of the International Food Policy Study, a repeated cross-sectional online survey of adults aged 18–100 years (*n* = 8435). Participants reported use of FoPNLs and NFTs, and five healthy eating efforts: consume less calories, saturated fat, sugar, sodium, and non-sugar sweeteners. Adjusted logistic regression models estimated associations between reported label use and engagement in any healthy eating effort, controlling for sociodemographic characteristics.

**Results:**

FoPNL use increased by 10.3 percentage points, and NFT use increased by 4.4 percentage points between 2019 and 2021. Participants who reported using NFTs and FoPNLs were more likely to engage in healthy eating efforts (OR range: 1.52–1.82). Associations between label use and healthy eating efforts did not differ by survey year.

**Conclusions:**

Reported use of FoPNL and NFT increased after 2020 policy changes in Mexico. The association between self-reported label use and self-reported healthy eating efforts did not differ between 2019 and 2021.

## Highlights

• Nutrition label use increased in Mexico after the 2020 policy change.
• Adults using Warning Labels were more likely to report healthy eating efforts.
• Adults using Nutrition Facts were more likely to report healthy eating efforts.
• No differences across years were observed between label use and healthy eating efforts.

## 1 Introduction

Healthy diets include appropriate caloric intake, higher consumption of unsaturated rather than saturated fats, and limited intake of highly processed foods or added sugars (Willett et al., 2019). However, the current food system promotes the production and consumption of ultraprocessed products (UPPs) (Pan American Health Organization, 2019). A previous study shows that 72% of food and beverage products in the Mexican market in 2017 were classified as UPPs, and 98% of these products have an excess in one or more nutrients of concern (sugar, sodium, saturated fat, trans fat) or calories (Cruz-Casarrubias et al., 2021). Greater UPP consumption has been associated with poorer dietary quality, including higher intake of added sugar, saturated fats, lower intake of protein and dietary fiber, and higher energy density, all associated with the development of non-communicable diseases (NCDs) (Pan American Health Organization, 2019). Public policies are needed to control and reduce the production, sale, promotion, availability and consumption of UPPs (Monteiro and Cannon, 2019). Government-mandated nutrition labeling can guide consumers toward informed food purchases and healthier eating choices by providing clear information about critical nutrients in packaged products (World Health Organization (WHO), 2019).

In Mexico, one of the earliest policy responses to address these challenges was the introduction of mandatory nutrition labeling. In 1994, the Mexican Official Standard NOM-051-SCFI-1994 required the inclusion of a nutrition facts table (NFT) on packaged food products (Diario Oficial de la Federación, 1996). In 2020, the NFT format was revised to provide clearer information to consumers (Diario Oficial de la Federación, 2020), including distinguishing between total sugars and added sugars (those added during the manufacturing process), standardizing serving sizes per 100 g or 100 ml, and requiring calorie information for the entire package. On a voluntary basis, industry may also provide nutrition information per serving.

To further support healthier food choices and modify the food environment, Mexico approved in 2015 the mandatory inclusion of front-of-package nutrition label (FoPNL) Guideline Dietary Amount (GDA) (Diario Oficial de la Federación, 2010). In October 2020, following the example of other Latin American countries, the GDA system was replaced with mandatory nutritional Warning Labels (WLs) (Diario Oficial de la Federación, 2020) to simplify information on the nutritional quality of packaged food products. The current WL system requires prominently placed black octagons on packaged products that exceed critical thresholds of calories, sugar, sodium, saturated fat, and trans-fat, using thresholds recommended by the Pan American Health Organization. Additionally, products containing caffeine or non-caloric sweeteners must display disclaimers with the statement “Avoid in Children” (Diario Oficial de la Federación, 2020).

Early evidence from before and immediately after the Mexican WL policy was implemented and that used diverse study designs (e.g., focus groups, surveys, and brief randomized experiments) suggests that WLs in Mexico may be more effective in fostering healthier food choices (Jáuregui et al., 2020) and are better understood and accepted (Vargas-Meza et al., 2019a; Oviedo-Solís et al., 2024) than the previous GDA system, including among low- and middle-income groups (Vargas-Meza et al., 2019b). During the Law Modification Period (2020-present), where the policy was implemented but not yet fully enforced, WLs showed promising results, including improving consumers' perceptions of unhealthy foods (Campos-Nonato et al., 2022), and contributing to self-reported decreases in the purchase of certain unhealthy foods among Mexican youth and adults (Contreras-Manzano et al., 2024).

FoPNLs may influence consumer purchase and intake behaviors through multiple pathways: (1) cognitive effects, such as altering perceptions of a product's healthfulness, challenging prior beliefs, or providing unexpected information; (2) triggering emotional responses, including concern or fear; and (3) behavioral reinforcement, by serving as a salient reminder of long-term health goal (Roberto et al., 2021). However, the effectiveness of labeling can vary depending on the type of labeling implemented. Among other aspects, the complexity of information presented, and the challenges in comparing products at the point of selection, may play a key role (Roberto et al., 2021). Studies have shown that WLs are more effective than GDAs in promoting healthier food choices, both in experimental studies assessing purchasing intentions and in real-world evaluations using sales data as behavioral outcomes (Jáuregui et al., 2020; Roberto et al., 2021). This greater effectiveness may arise through multiple pathways, including deliberate processing of label information as well as more rapid, heuristic processing supported by the simple, salient, and interpretative design of WL systems, which perform well among lower literacy and numeracy groups (Constantin et al., 2021).

Although substantial evidence demonstrates that nutrition labels influence consumer behavior and using them is associated with better diets (Anastasiou et al., 2019), it is unclear whether people who consciously report using nutrition labels also report broader healthy eating efforts to reduce nutrients, ingredients, and calories displayed on nutrition labels and more wide-ranging impacts on healthy eating efforts. The objective of the present study was to examine the associations between self-reported use of nutrition labels and self-reported healthy eating efforts among Mexican adults, and to test whether these associations were stronger after the replacement of GDAs with WLs.

## 2 Methods

### 2.1 Study design and population

Data were analyzed from the 2019 and 2021 Mexico administration of the International Food Policy Study, a repeat cross-sectional online survey conducted among adults (18–100 years). Data from the 2020 survey were excluded because the Nutrition Labeling Policy was not yet fully implemented and because COVID-19 may have influenced dietary behaviors (Acton et al., 2022). Online, self-administered surveys were conducted in November–December in 2019 and 2021 with 4314 and 4172 Mexican respondents, respectively. Of these, 51 (0.6%) were excluded because of incomplete information on health dietary efforts or use of FoPNLs and NFT, leaving an analytical sample size of 4288 in 2019 and 4147 in 2021.

Participants were recruited from the Nielsen Consumer Insights Global Panel and its partner panels. Invitations with unique survey access links were sent to a random sample of panelists after demographic targeting (≥18 years, and resided in Mexico); panelists known to be ineligible were not invited. Potential respondents were screened for eligibility (≥18 years), and quotas for age and sex were applied to facilitate recruitment of a diverse sample that approximated known proportions in Mexico for males and females in four age groups: 18–29, 30–44, 45–64, and 65+. After screening, potential respondents were provided with information about the study and provided consent before participating. Respondents were compensated according to their panel's usual incentive structure, which included points-based or cash rewards redeemable for e-gift cards, catalog items, cash, donations, and/or chances to win monthly prizes. The survey was conducted in Spanish, and the average time to complete the surveys was 44 and 48 min for 2019 and 2021, respectively.

Post-stratification sample weights were constructed based on national population estimates by age, sex at birth, region, and ethnicity (Hammond et al., 2021; Hammond et al., 2023), and rescaled for the analytical sample size.

The study was reviewed and received ethics clearance by the University of Waterloo Research Ethics Committee (ORE # 30829) and the Research Ethics Committee of the National Institute of Public Health in Mexico.

### 2.2 Measures

#### 2.2.1 Reported healthy eating efforts

Participants were queried about their efforts in the past 12 months to consume more or less of certain foods and nutrients. For the purposes of this study, we consider the following: calories, saturated fat, sugar, sodium, and non-sugar sweeteners (stevia and aspartame) because the labeling policy in Mexico specifically focused on these nutrients or ingredients. Participants were asked, “Have you made an effort to consume more or less of the following in the past year?” with response options “consume less”, coded as 1, and “consume more”, “make no effort” or “don't know”, coded as 0. Response option “Refuse to answer” were coded as missing. These binary data were recoded as reported engaging in any healthy eating efforts (number of efforts ≥1) and no reported efforts (number of efforts =0), expressed as a binary dependent variable.

#### 2.2.2 Self-reported use of nutrition labels

Use of the NFT and FoPNLs were assessed by asking, “How often do you use this type of food label when deciding to buy a food product?” while being shown a picture of an NFT or FoPNL (GDA and WL images were shown in 2019 and 2021, respectively). Response options used a 5-point scale, with responses recoded (0 = never/rarely/sometimes and 1 = often/all the time). Respondents who answered “Don't know” or reported that they had not seen the NFT or FoPNL were coded as “0.”

#### 2.2.3 Covariates

Sociodemographic characteristics included in the analyses were sex at birth (female or male); age group (18–29, 30–44, 45–59, and ≥ 60 years); education based on the last grade that participants completed in school: 1) high school or less (none, preschool, primary, secondary, high school, or baccalaureate, normal basic) 2) technical studies (technical or commercial studies with completed primary, technical or commercial studies with completed secondary, technical or commercial studies with completed high school) and 3) undergraduate or more (bachelor's degree, bachelor/professional, master's degree or doctorate). Regions of residence in Mexico (north, south, center, and Mexico City); and ethnicity (non-indigenous and indigenous). Also, participants were asked about the presence of children (under 18 years) in household (yes/no) and about perceived income adequacy by asking, “Thinking about your total monthly income, how difficult or easy is it for you to make ends meet?” (very difficult/difficult/neither easy nor difficult/easy/very easy) (Hammond et al., 2022).

Health literacy status was measured using an adapted, text version of the Newest Vital Sign. This measure consisted of six questions about information on the Mexican NFT of a carton of ice cream that assessed their ability to make mathematical calculations, read and apply label information, and understand label information (Hammond et al., 2022). A score between 0 and 6 was calculated based on the number of correct answers, with higher scores corresponding greater health literacy. Participants were classified as 1 “high likelihood of limited literacy” (score 0–1), 2 “possibility of limited literacy” (score 2–3), and 3 “adequate literacy” (score 4–6), as per previously employed methods (Bhawra et al., 2023a). Response options “Refuse to answer” and “Don't know” were coded as missing.

### 2.3 Statistical analysis

Descriptive statistics summarized sociodemographic characteristics for weighted samples. Pearson χ^2^ tests examined differences in sociodemographic characteristics by survey year.

Logistic regression models, stratified by year and adjusting for covariates (see above), were used to estimate associations between the use of FoPNLs (GDA in 2019 and WL in 2021) and NFTs with engagement in any healthy eating efforts, treated as a binary outcome. To assess whether these associations differed by survey year, interaction terms between survey year and FoPNLs or NFTs use were added to the fully adjusted models. Participants with missing data on the exposure, outcome, or covariates required for each model were excluded from that analysis (i.e., *n* = 109 or 2.54% excluded for 2019; *n* = 136 or 3.28% excluded for 2021). The F-test was used to test for statistical significance (*p* < 0.01). Analyses were performed using Stata v18.

## 3 Results

Table 1 describes the participant characteristics. In the weighted sample of 8435 participants, more than 52.30% were female, more than 60% had undergraduate studies or more, and 79.85% self-identified as non-indigenous. More than 60% reported making efforts to consume less sugar, saturated fat, and non-sugar sweeteners in 2019 and 2021. The proportion of participants making at least one effort was lower in 2021 (83.43%) than in 2019 (88.11%) (Table 1).

**Table 1:** Weighted characteristics of participants from the International Food Policy Study 2019 and 2021.

	Total	2019	2021	
	n(%)	n(%)	n(%)	*P*-value
N	8435 (100.0%)	4288 (50.8%)	4147 (49.2%)	
				
Sex at birth				
Male	4024 (47.70%)	2041 (47.60%)	1983 (47.82%)	0.86
Female	4411 (52.30%)	2247 (52.40%)	2164 (52.18%)	
				
Age				
18–29 years	2467 (29.24%)	1297 (30.24%)	1170 (28.21%)	0.01
30–44 years	2658 (31.51%)	1381 (32.20%)	1277 (30.79%)	
45–59 years	2364 (28.03%)	1188 (27.71%)	1176 (28.36%)	
≥60 years	946 (11.22%)	422 (9.85%)	524 (12.64%)	
				
Education level completed a				
High school or less	1798 (21.35%)	967 (22.60%)	831 (20.05%)	0.02
Technical studies	1162 (13.80%)	556 (12.99%)	606 (14.64%)	
Undergraduate or more	5462 (64.85%)	2756 (64.40%)	2706 (65.32%)	
				
Regions of residence in Mexico b				
North	2009 (23.82%)	1012 (23.60%)	997 (24.05%)	0.92
South	2445 (28.99%)	1253 (29.22%)	1192 (28.75%)	
Centre	3280 (38.89%)	1662 (38.77%)	1618 (39.01%)	
Mexico City	700 (8.30%)	361 (8.41%)	339 (8.19%)	
				
Perceived income adequacy c				
Very difficult	846 (10.10%)	448 (10.50%)	398 (9.70%)	0.23
Difficult	2589 (30.92%)	1350 (31.64%)	1239 (30.16%)	
Neither easy nor difficult	3398 (40.58%)	1713 (40.16%)	1685 (41.02%)	
Easy	1202 (14.36%)	602 (14.11%)	600 (14.62%)	
Very easy	338 (4.04%)	153 (3.59%)	185 (4.50%)	
				
Health literacy d				
High likelihood of limited literacy	3108 (36.88%)	1445 (33.74%)	1663 (40.13%)	<0.01
Possibility of limited literacy	2561 (30.40%)	1305 (30.48%)	1256 (30.31%)	
Adequate literacy	2757 (32.72%)	1533 (35.78%)	1225 (29.56%)	
				
Ethnicity e				
Indigenous	1666 (20.15%)	892 (21.17%)	774 (19.09%)	0.07
Non-indigenous	6604 (79.85%)	3321 (78.83%)	3283 (80.91%)	
			
Presence of children (under 18 years) in household				
No children in household	4175 (49.54%)	2055 (47.96%)	2120 (51.18%)	0.01
Children in household (under 18 years)	4252 (50.46%)	2230 (52.04%)	2022 (48.82%)	
				
Reported healthy eating efforts f				
Calories	3876 (45.96%)	2056 (47.94%)	1821 (43.90%)	<0.01
Sugar	5320 (63.07%)	2797 (65.23%)	2523 (60.85%)	<0.01
Saturated fat	5453 (64.65%)	2860 (66.71%)	2593 (62.53%)	<0.01
Sodium	5101 (60.47%)	2665 (62.15%)	2436 (58.73%)	0.01
Non-sugar sweeteners g	5251 (62.26%)	2739 (63.88%)	2512 (60.58%)	0.01
				
Number of reported healthy eating efforts				
None healthy efforts	1197 (14.19%)	510 (11.89%)	687 (16.57%)	<0.01
One healthy effort	875 (10.38%)	423 (9.86%)	452 (10.91%)	
Two healthy efforts	975 (11.56%)	524 (12.22%)	451 (10.88%)	
Three healthy efforts	1421 (16.85%)	756 (17.63%)	666 (16.05%)	
Four healthy efforts	1919 (22.75%)	999 (23.30%)	920 (22.19%)	
Five healthy efforts	2047 (24.27%)	1077 (25.11%)	970 (23.40%)	

a Participants were asked, “What is the last year and grade you completed at school?” 1) high school or less (none, preschool, primary, secondary, high school, or baccalaureate, normal basic) 2) Technical studies (technical or commercial studies with completed primary, technical, or commercial studies with completed secondary, technical or commercial studies with completed high school) and 3) Undergraduate or more (bachelor's degree, bachelor/professional, master's degree or doctorate)

b North region: Baja California, Baja California Sur, Chihuahua, Coahuila, Durango, Nuevo León, Sinaloa, Sonora, Tamaulipas and Zacatecas. South region: Campeche, Chiapas, Guerrero, Morelos, Oaxaca, Puebla, Quintana Roo, Tabasco, Veracruz, and Yucatán. Centre region: Aguascalientes, Colima, Guanajuato, Hidalgo, Jalisco, Mexico, Michoacán, Nayarit, Querétaro, San Luis Potosí, and Tlaxcala. Mexico City

c Participants were asked: “Thinking about your total monthly income, how difficult or easy is it for you to make ends meet?

d Health literacy: High likelihood of limited literacy (score 0–1), Possibility of limited literacy (score 2–3), Adequate literacy (score 4–6)

e Participants were asked, “According to your culture, are you considered indigenous?”

f Reported healthy eating efforts: Efforts to consume less nutrients, ingredients, or calories related to labeled

g Consume less ‘Artificial’ low calorie sweeteners like aspartame or ‘Natural’ low-calorie sweeteners like stevia

Between 2019 and 2021, the percentage of participants who reported using FoPNLs increased by 10.31 percentage points (pp) (*p* value <0.01), rising from 36.20% for GDAs to 46.51% for WLs, while those who reported using NFT increased by 4.34 pp. from 39.25% to 43.59% (*p* value <0.01) (Fig. 1).

**Fig. 1:**
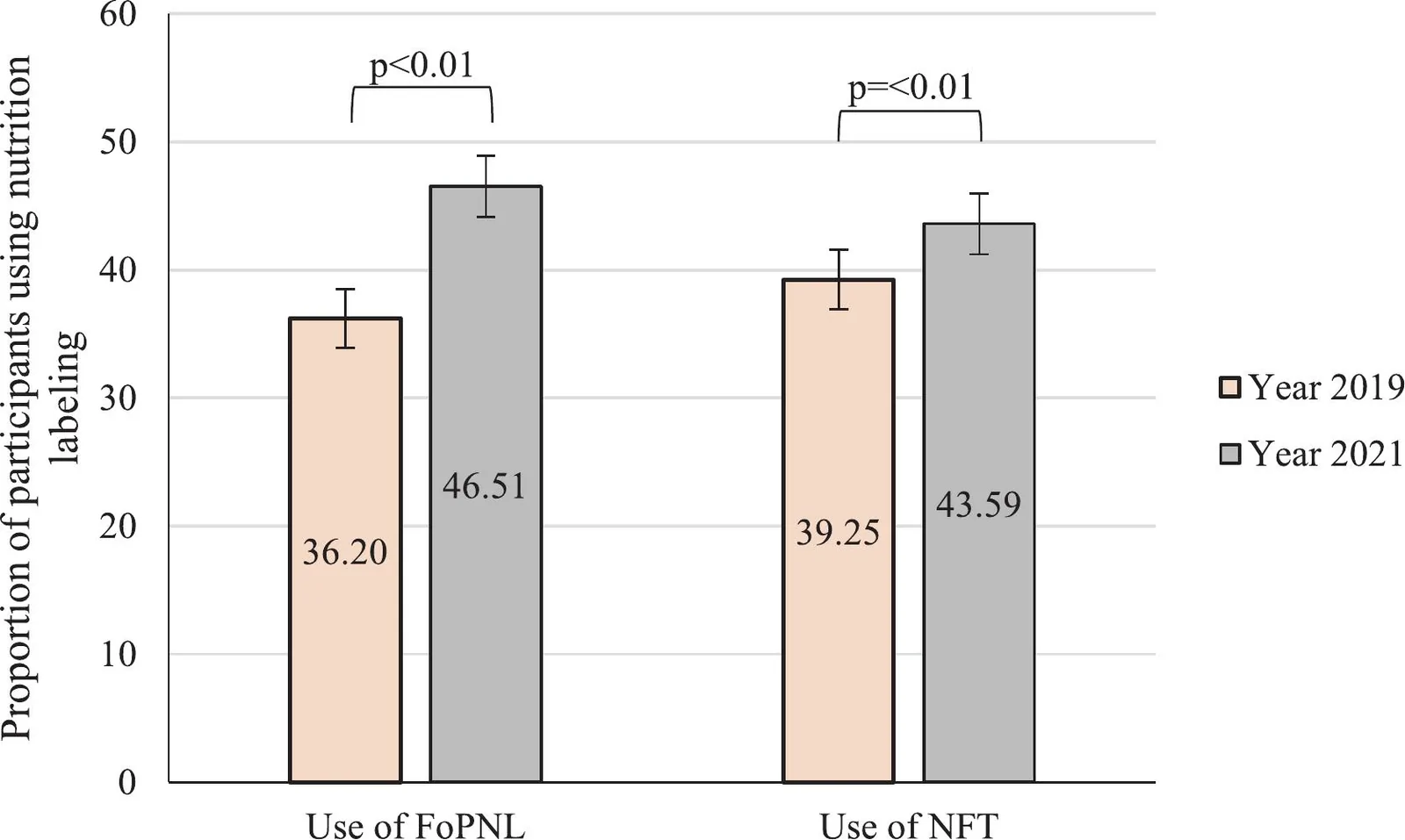
Adjusted percentage (%) of participants using nutrition labeling before and after nutrition labeling policy changes in Mexico, International Food Policy Study 2019–2021. Percentage of participants using nutrition labeling derived from logistic regression models adjusted for sex at birth, age, education level completed, regions of residence in Mexico, perceived income adequacy, health literacy, ethnicity, and presence of children (under 18 years) in household. Percentages were estimated using margins. FoPNL: Front-of-package nutrition label implemented in 2019 was Guideline Daily Amount (GDA) and 2021 was Warning Label (WL). NFT: Nutrition Fact Table.

As shown in Table 2, participants who reported using NFT in 2019 and 2021 were more likely to report engaging in any type of healthy eating effort (odds ratio (OR):1.82, 99%CI: 1.17–2.82 and OR: 1.70, 99%CI: 1.25–2.30, respectively), than participants who did not use NFTs (Table 2). Similar associations were observed in 2021 between using WLs (OR: 1.52, 99%CI: 1.12–2.04), while in 2019, when GDAs were used for FoPNLs, the association was not statistically significant (OR: 1.52, 99%CI: 0.97–2.39).

**Table 2:** Weighted estimates of associations between reported any healthy eating efforts and use of FoPNLs and NFT among Mexican adults, 2019 and 2021.

		Reported any healthy eating efforts (vs no effort)^a^, ^b^	
		2019	2021
		adjusted OR (99%CI)	adjusted OR (99%CI)
Use of FoPNL^c^	No	1	1
	Yes	1.52 (0.97, 2.39)	1.52 (1.12, 2.04)
			
Use of NFT d	No	1	1
	Yes	1.82 (1.17, 2.82)	1.70 (1.25, 2.30)

a Reported healthy eating efforts: Efforts to consume less nutrients, ingredients, or calories related to labeled

b Logistic regression adjusted for sex at birth, age, education level completed, regions of residence in Mexico, perceived income adequacy, health literacy, ethnicity, and presence of children (under 18 years) in household.

c FoPNL: Front-of-package nutrition label implemented in 2019 was Guideline Daily Amount (GDA) and 2021 was Warning Label (WL).

d NFT: Nutrition Fact Table.

Interaction terms between survey year and the use of FoPNLs (Interaction term = 0.96, 99% CI: 0.62, 1.49, *p* = 0.82) or NFTs (Interaction term = 0.92, 99% CI: 0.60, 1.42, *p* = 0.64) were not statistically significant, indicating that the association between label use and healthy eating efforts were consistent across survey years.

## 4 Discussion

This is the first study to examine associations between self-reported use of nutrition labels and reported healthy eating efforts, including both FoPNLs and NFTs. Overall, results suggest that adults who reported frequently using these labels increased after the labeling policy update; furthermore, these people were more likely to say they engaged in healthy eating efforts than those who never or sometimes reported using the labels. Although the association between label use and self-reported healthy eating efforts was statistically significant for WL use (i.e., 2021) but not for GDA use (i.e., 2019), the interaction model results indicated that the strength of association was similar across years. This suggests that the shift from GDAs to WLs did not alter the overall relationship between self-reported, conscious label use and reported healthy eating efforts, and that adults who consciously reported using either labeling system were similarly likely to report healthy eating efforts.

The relationship between self-reported label use and reported healthy eating efforts is likely bidirectional: individuals who are already trying to eat a healthier diet may be more likely to be more conscious of using labels, while well-designed labels may encourage a larger share of consumers to try to eat a healthier diet (Shangguan et al., 2019; Acton et al., 2023). Recognizing this dual relationship is particularly important in countries like Mexico, where the prevalence of overweight and obesity is over 70% (Campos-Nonato et al., 2023a) and other diet-related NCDs, such as hypertension, affect around 30% of the population (Campos-Nonato et al., 2023b).

Our findings also suggest that, contrary to the authors' hypothesis, there was no significant difference in reported engaging in healthy eating efforts between those who reported using GDAs in 2019, WLs in 2021, or NFTs in both years, compared with those who did not use these labels. This was unexpected, given evidence that WLs can promote healthier food choices more quickly (Jáuregui et al., 2020; Oviedo-Solís et al., 2024; Sagaceta-Mejía et al., 2022) and have greater levels of acceptance (Oviedo-Solís et al., 2024) and understanding (Vargas-Meza et al., 2019a; Campos-Nonato et al., 2022) compared to GDA. Consistent with prior studies (Acton et al., 2023), our results show that WLs are now used by a greater proportion of Mexican adults than from before WL implementation, when GDA was the required FoPNL. This shift coincides with a slowing of the growth in overweight and obesity rates among the Mexican population, following broader prevention efforts, including the implementation of a tax on sugar-sweetened beverages in 2014 and the introduction of WLs in 2020 (Barquera et al., 2024). However, greater self-reported use of WLs did not appear to translate into higher reported healthy eating efforts compared to self-reported GDA users.

One possible explanation for these findings is that our surveys only captured self-reported, conscious use of nutrition labels, and measures only assessed conscious healthy eating efforts. Although WLs have been shown to be more effective than GDA in promoting healthier food choices, part of this advantage may arise from their simple and salient design (Bhawra et al., 2023b), which facilitates more rapid or heuristic processing that may not be captured by self-reported label use (Bhawra et al., 2023b). Therefore, WLs may be more effective when people are not making conscious efforts because they allow people to make more unconscious, heuristically motivated food choices. Furthermore, the implementation of WLs in Mexico prompted the reformulation of numerous products (Salgado et al., 2025). As a result, even in the absence of conscious changes in eating behaviors, consumers may experience improvements in the nutritional quality of their diets through the consumption of reformulated products. In addition, a ceiling effect may exist. Since label users already report greater engagement in healthy eating, WLs may not further increase this measure, even if they improve comprehension. Another explanation relates to how participants interpret ‘healthy eating efforts’. For those beginning dietary modifications, such ‘efforts’ may represent conscious actions to avoid specific nutrients or ingredients known to be linked to NCDs. In contrast, individuals with established healthy eating routines may not view these choices as active efforts, as they have become habitual and non-conscious (Roberto et al., 2021; Hollands et al., 2016). These factors could explain why we observed no significant differences in the association between reported healthy eating efforts and WL and GDA users, even though use of WLs was more frequently reported than for GDAs (Oviedo-Solís et al., 2024).

In addition, the results indicated that use of NFT was associated with reported healthy eating efforts among Mexican adults, with a magnitude similar to that observed with FoPNLs. Other studies suggest that regular NFT use is associated with healthier dietary intakes (Christoph et al., 2018; Restrepo, 2025) and consumers' understanding of the NFTs directly influences their use (Huang et al., 2022). In addition, the observed association of similar magnitude in our results between the use of any label and reported healthy eating efforts may be because people who use nutrition labels share positive attitudes about healthy diets, beliefs about the importance of nutrition labels in guiding food selection, self-efficacy related to eating behavior, and nutrition knowledge and education (Christoph et al., 2016).

These results generally support the use of FoPNLs and NFT as public health policy tools that are associated with healthy decision-making. This raises questions about the extent to which labeling policies can actively change consumer behaviors versus reflecting existing dietary habits. Further investigation is required to elucidate the influence of nutrition labeling policy on changes in diet-related behavior in the Mexican population and the impact on long-term health outcomes.

### 4.1 Strengths and limitations

Strengths of this study include the measurement of healthy eating efforts; and use of FoPNLs and NFTs before and after the implementation of new labeling regulations in Mexico, and the large sample size of Mexican adults. However, some limitations should be acknowledged, including those common to survey research. Respondents were recruited using non-probability-based sampling; therefore, the findings do not provide nationally representative estimates. For example, although the data were weighted by age group, sex, region, and ethnicity, the Mexico sample had notably higher levels of education than the national estimates. However, covariates that can be potentially related to use of FoPNLs and NFTs were controlled for in all models.

## 5 Conclusions

Results from this study show that the reported use of FoPNLs and NFTs increased after 2020 policy changes in Mexico. However, the association between self-reported label use and self-reported healthy eating efforts did not differ between 2019 and 2021, suggesting that adults who consciously reported using nutrition labels were similarly likely to report healthy eating efforts under both labeling systems.

## Declaration of generative AI and AI-assisted technologies in the manuscript preparation process

The declaration does not apply because it was used as a basic tool, such as tools used to check grammar, spelling and references.

## Funding acknowledgment

Funding for the International Food Policy Study was provided by a 10.13039/501100000024Canadian Institutes of Health Research (CIHR) Project Grant (PJT-162167), with additional support for the adult survey from the National Institute of Diabetes and Digestive and Kidney Disorders of the 10.13039/100000002National Institutes of Health (R01 DK128967), and the 10.13039/501100000272National Institute for Health Research (NIHR13059). The funders had no role in the study design, collection, analysis, and interpretation of the data, writing of the report, or the decision to submit the article for publication.

The Ministry of Science, Humanities, Technology, and Innovation (SECIHTI) partially supported CIOS's time writing the paper. The authors thank Jennifer Mandelbaum, Indira Gutierrez Orozco and Simón Barquera for their assistance with manuscript review and editing.

## CRediT authorship contribution statement

**Cecilia I. Oviedo-Solís:** Writing – original draft, Visualization, Formal analysis. **Kathia L. Quevedo:** Writing – review & editing, Visualization. **Alejandra Jauregui:** Writing – review & editing, Visualization. **Alejandra Contreras:** Writing – review & editing. **Lana Vanderlee:** Writing – review & editing, Methodology, Conceptualization. **Rachel E. Davis:** Writing – review & editing, Funding acquisition. **James F. Thrasher:** Writing – review & editing, Funding acquisition, Conceptualization. **David Hammond:** Writing – review & editing, Supervision, Methodology, Funding acquisition, Conceptualization.

## Ethics declaration

### Informed consent and patient details

Written informed consent to take part in the study and to publish the article has been obtained from all participants or their legal representatives. The privacy rights of participants have been observed.

### Studies in Human

This study was performed in compliance with relevant laws, regulatory frameworks and guidelines where the research took place.

This study was approved by the University of Waterloo Research Ethics Committee and Research Ethics Committee of the National Institute of Public Health in Mexico.

(Approval No. ORE # 30829, CI: 1641).

## Declaration of competing interest

The authors declare that they have no known competing financial interests or personal relationships that could have appeared to influence the work reported in this paper.

## Data Availability

Data will be made available on request.

## References

[bb0005] Acton R.B., Vanderlee L., Cameron A.J. (2022). Self-reported impacts of the COVID-19 pandemic on diet-related behaviors and food security in 5 countries: results from the international food policy study 2020. J. Nutr..

[bb0010] Acton R.B., Rynard V.L., Adams J. (2023). Awareness, use and understanding of nutrition labels among adults from five countries: findings from the 2018–2020 international food policy study. Appetite.

[bb0015] Anastasiou K., Miller M., Dickinson K. (2019). The relationship between food label use and dietary intake in adults: a systematic review. Appetite.

[bb0020] Barquera S., Hernández-Barrera L., Oviedo-Solís C. (2024). Obesidad en adultos. Salud Publica Mex..

[bb0025] Bhawra J., Kirkpatrick S.I., Hall M.G., Vanderlee L., White C.M., Hammond D. (2023). Patterns and correlates of nutrition knowledge across five countries in the 2018 international food policy study. Nutr. J..

[bb0030] Bhawra J., Kirkpatrick S.I., Hall M.G. (2023). A five-country study of front- and back-of-package nutrition label awareness and use: patterns and correlates from the 2018 international food policy study. Public Health Nutr..

[bb0035] Campos-Nonato I., Cervantes-Armenta M.A., Pacheco-Miranda S. (2022). Perception and understanding of guideline daily amount and warning labeling among Mexican adults during the law modification period. Nutrients.

[bb0040] Campos-Nonato I., Galván-Valencia Ó., Hernández-Barrera L., Oviedo-Solís C., Barquera S. (2023). Prevalencia de obesidad y factores de riesgo asociados en adultos mexicanos: resultados de la Ensanut 2022. Salud Publica Mex..

[bb0045] Campos-Nonato I., Oviedo-Solís C., Vargas-Meza J. (2023). Prevalencia, tratamiento y control de la hipertensión arterial en adultos mexicanos: resultados de la Ensanut 2022. Salud Publica Mex..

[bb0050] Christoph MJ, An R, Ellison B. Correlates of nutrition label use among college students and young adults: a review. Public Health Nutr. Cambridge University Press. 2016;19(12):2135–2148. doi:10.1017/S1368980015003183. PMC10271094 26549218

[bb0055] Christoph M.J., Larson N., Laska M.N., Neumark-Sztainer D. (2018). Nutrition facts panels: who uses them, what do they use, and how does use relate to dietary intake?. J. Acad. Nutr. Diet..

[bb0060] Constantin A., Cabrera O.A., Ríos B. (2021). A human rights-based approach to non-communicable diseases: mandating front-of-package warning labels. Glob. Health.

[bb0065] Contreras-Manzano A., White C.M., Nieto C. (2024). Self-reported decreases in the purchases of selected unhealthy foods resulting from the implementation of warning labels in Mexican youth and adult population. Int. J. Behav. Nutr. Phys. Act..

[bb0070] Cruz-Casarrubias C., Tolentino-Mayo L., Vandevijvere S., Barquera S. (2021). Estimated effects of the implementation of the Mexican warning labels regulation on the use of health and nutrition claims on packaged foods. Int. J. Behav. Nutr. Phys. Act..

[bb0075] Diario Oficial de la Federación. *Norma Oficial Mexicana NOM-051-SCFI-1994*. 1996.

[bb0080] Diario Oficial de la Federación. *Norma Oficial Mexicana NOM-051-SCFI/SSA1–2010*. 2010.

[bb0085] Diario Oficial de la Federación. *Modificación a La Norma Oficial Mexicana NOM-051-SCFI/SSA1–2010*. 2020. https://www.dof.gob.mx/2020/ SEECO/NOM_051.pdf.

[bb0090] Hammond D., White C., Rynard V., Vanderlee L. (2021). International Food Policy Study: Technical Report - 2019 Survey (Wave 3). http://www.foodpolicystudy.com/methods.

[bb0095] Hammond D., Vanderlee L., White C.M. (2022). The conceptual framework for the international food policy study: evaluating the population-level impact of food policy. J. Nutr..

[bb0100] Hammond D., White C., Rynard V., Vanderlee L. (2023). International Food Policy Study: Technical Report– 2021 Survey (Wave 5). http://www.foodpolicystudy.com/methods.

[bb0105] Hollands G.J., Marteau T.M., Fletcher P.C. (2016). Non-conscious processes in changing health-related behaviour: a conceptual analysis and framework. Health Psychol. Rev..

[bb0110] Huang Z., Li H., Huang J. (2022). Determinants of nutrition facts table use by Chinese consumers for nutritional value comparisons. Int. J. Environ. Res. Public Health.

[bb0115] Jáuregui A., Vargas-Meza J., Nieto C. (2020). Impact of front-of-pack nutrition labels on consumer purchasing intentions: a randomized experiment in low- and middle-income Mexican adults. BMC Public Health.

[bb0120] Monteiro C.A., Cannon G.J. (2019). The role of the transnational ultra-processed food industry in the pandemic of obesity and its associated diseases: problems and solutions. World Nutr..

[bb0125] Oviedo-Solís C.I., Tolentino-Mayo L., Quevedo K.L. (2024). Impact of front-of-package nutrition labels on acceptability and objective understanding: a randomized experiment in Latin American adults. Appetite.

[bb0130] Pan American Health Organization (2019). Ultra-Processed Food and Drink Products in Latin America: Sales*,* Sources*,* Nutrient Profiles and Policy Implications. http://iris.paho.org/xmlui/bitstream/handle/123456789/51094/9789275120323_eng.pdf?sequence=5&isAllowed=y.

[bb0135] Restrepo B.J. (2025). Who regularly uses the nutrition facts label? Exploring demographic, socioeconomic, and geographic Differences. http://www.ers.usda.gov.

[bb0140] Roberto C.A., Ng S.W., Ganderats-Fuentes M. (2021). The influence of front-of-package nutrition labeling on consumer behavior and product reformulation. Annu. Rev. Nutr..

[bb0145] Sagaceta-Mejía J., Tolentino-Mayo L., Cruz-Casarrubias C., Nieto C., Barquera S. (2022). Understanding of front of package nutrition labels: guideline daily amount and warning labels in Mexicans with non-communicable diseases. PLoS One.

[bb0150] Salgado J.C., Pedraza L.S., Contreras-Manzano A., Aburto T.C., Tolentino-Mayo L., Barquera S. (2025). Product reformulation in non-alcoholic beverages and foods after the implementation of front-of-pack warning labels in Mexico. PLoS Med..

[bb0155] Shangguan S., Afshin A., Shulkin M. (2019). A Meta-analysis of food labeling effects on consumer diet behaviors and industry practices. Am. J. Prev. Med..

[bb0160] Vargas-Meza J., Jáuregui A., Contreras-Manzano A. (2019). Acceptability and understanding of front-of-pack nutritional labels: an experimental study in Mexican consumers. BMC Public Health.

[bb0165] Vargas-Meza J., Jáuregui A., Pacheco-Miranda S., Contreras-Manzano A., Barquera S. (2019). Front-of-pack nutritional labels: understanding by low- and middle-income Mexican consumers. PloS One.

[bb0170] Willett W., Rockström J., Loken B. (2019). Food in the Anthropocene: the EAT–lancet commission on healthy diets from sustainable food systems. Lancet.

[bb0175] World Health Organization (WHO) (2019). Guiding Framework Principles and Labelling for Front-of-Pack Manual for Promoting Healthy Diet.

